# Collaborative care for anxiety disorders in primary care: a systematic review and meta-analysis

**DOI:** 10.1186/s12875-016-0466-3

**Published:** 2016-06-02

**Authors:** Anna DT Muntingh, Christina M van der Feltz-Cornelis, Harm WJ van Marwijk, Philip Spinhoven, Anton JLM van Balkom

**Affiliations:** Department of Psychiatry, VU University Medical Center / GGZ inGeest, A.J. Ernststraat 1187, Amsterdam, 1081 HL The Netherlands; Faculty of Social Sciences, Tranzo department, Tilburg University, PO Box 90153, Tilburg, 5000 LE The Netherlands; Top Clinical Centre for Body, Mind and Health, GGZ Breburg, Lage Witsiebaan 4, Tilburg, 5042 DA The Netherlands; Centre for Primary Care, Institute for Population Health, University of Manchester, Manchester, UK; Department of General Practice and Elderly Care Medicine and Institute for Health and Care Research (EMGO+), VU University Medical Centre, Van der Boechorststraat 7, Amsterdam, 1081BT The Netherlands; Institute of Psychology, Leiden University, PO Box 9555, Leiden, 2300 RB The Netherlands; Department of Psychiatry, Leiden University Medical Centre, PO Box 9600, Leiden, 2300 RC The Netherlands

**Keywords:** Collaborative care, Primary care, Anxiety disorders, Systematic review, Meta-analysis, Randomized controlled trials

## Abstract

**Background:**

Studies evaluating collaborative care for anxiety disorders are recently emerging. A systematic review and meta-analysis to estimate the effect of collaborative care for adult patients with anxiety disorders in primary care is therefore warranted.

**Methods:**

A literature search was performed. *Data sources:* PubMed, Psycinfo, Embase, Cinahl, and the Cochrane library. *Study eligibility criteria:* Randomized controlled trials examining the effects of collaborative care for adult primary care patients with an anxiety disorder, compared to care as usual or another intervention. *Synthesis methods:* Standardized mean differences (SMD) on an anxiety scale closest to twelve months follow-up were calculated and pooled in a random effects meta-analysis.

**Results:**

Of the 3073 studies found, seven studies were included with a total of 2105 participants. Included studies were of moderate to high quality. Collaborative care was superior to care as usual, with a small effect size (SMD = 0.35 95 % CI 0.14–0.56) for all anxiety disorders combined and a moderate effect size (SMD = 0.59, 95 % CI 0.41–0.78) in a subgroup analysis (five studies) on patients with panic disorder.

**Conclusions:**

Collaborative care seems to be a promising strategy for improving primary care for anxiety disorders, in particular panic disorder. However, the number of studies is still small and further research is needed to evaluate the effectiveness in other anxiety disorders.

## Background

Anxiety disorders constitute the most prevalent category of psychiatric disorders [1]. Anxiety disorders have a negative impact on quality of life and are associated with significant healthcare- and productivity costs [2]. Adults with an anxiety disorder mainly receive care in primary care [3–5]. In many countries however, the quality of care for adults with anxiety disorders leaves room for improvement [4, 6]. Although clinical guidelines recommend cognitive behavioral therapy (CBT) or antidepressant medication as the treatment of choice in primary care, these evidence-based treatments are not often adequately applied in primary care [7].

Several barriers still exist in providing evidence-based care for anxiety disorders in primary care, which may be related to patient characteristics, provider characteristics or the organizational context of primary care [8–11]. Therefore, multifaceted interventions that focus on patient, provider and organization of care have been proposed as the most promising strategy to improve primary mental healthcare [12, 13]. Collaborative care models are such multifaceted interventions, bringing mental health expertise into primary care by introducing new members into the primary care team. Typically, this new member is a “care manager” - a mental health professional who coordinates care, provides evidence-based interventions, and actively monitors the patients’ symptoms [14]. The care manager usually is a non-physician professional, such as a psychologist, a social worker or a psychiatric nurse. The care manager works in close collaboration with the primary care physician and preferably providers have access to the tailored advice of a psychiatrist. Collaborative care models often vary in types of interventions used, the intensity of treatment, collaboration, and follow-up [15]. However, some essential elements have been described by experts and in systematic reviews, which consider cooperation between the primary care physician and at least one other professional, provision of evidence-based treatment, and active monitoring of symptoms [14, 16–18]. Interventions or organizational models similar to collaborative care are sometimes referred to as integrated care, enhanced care, or care management.

### Rationale

Previous reviews on collaborative care included only a limited number of studies on anxiety disorders (4 in the review of Archer and colleagues [19] and 3 in the review of Woltmann and colleagues [20]). Furthermore, all of these studies were conducted in the United States, which may limit generalizability to primary care in other countries. We are furthermore aware of recent studies [21, 22] in other countries, so we systematically reviewed the literature on collaborative (primary) care for anxiety disorders, again.

Evidence of the effectiveness of collaborative care in the treatment of depression is well established and was reviewed thoroughly in several meta-analyses [19, 20, 23, 24]. The field has progressed to identifying elements of collaborative care that contribute most to its effectiveness. Recently, Coventry and colleagues [25] concluded that the provision of a psychological intervention increases the effectiveness of collaborative care based on a meta-regression. We were curious to what extent and how this could also apply to anxiety disorders.

### Objectives

We performed a systematic review and meta-analysis to summarize results from randomized controlled trials about the effectiveness of collaborative care for anxiety disorders in adult primary care patients compared to care as usual. In addition, we evaluated the effects for specific anxiety disorders and the influence of several characteristics of study procedures and collaborative care interventions on the effectiveness of the collaborative care model. We used the PRISMA checklist for reporting systematic reviews [26].

## Methods

### Eligibility criteria

#### Design and population

We included published, randomized controlled trials (RCTs) that evaluated collaborative care compared to care as usual or another active intervention in adult primary care patients with an anxiety disorder and that reported outcomes on a standardized scale for anxiety severity. Both individually randomized and cluster randomized trials were included.

Studies had to include adult (>18 years) subjects recruited in a primary care setting with an anxiety disorder as established with a valid diagnostic interview, according to research diagnostic criteria, or with a cut-off score on a validated scale. Comorbid medical or psychiatric conditions were allowed, as long as the intervention focused on the anxiety disorder.

#### Intervention and comparison intervention

Collaborative care interventions were defined by the application of criterion 1 in combination with criterion 2 and/or 3 [14, 16–18]:

1. The primary care physician is supported by at least one other professional with a different field of expertise (e.g. care manager, consultant psychiatrist), and they work together in providing care for the patient.
2. Evidence-based treatment is provided.
3. Process and outcome of treatment is monitored.

Studies evaluating the provision of services by an on-site mental health professional were excluded, unless reference was made to enhanced collaboration between the primary care physician and the mental health professional. Collaboration between professionals had to be ensured by team meetings, consultation or supervision, or a digital communication system. The collaborative care intervention could be compared to care as usual, a waitlist condition, or another active intervention.

#### Outcomes

Studies that reported outcomes at follow-up closest to 12 months on a validated continuous anxiety scale or dichotomous interview (indicating response or remission) were included. Standardized scales or interviews could measure general anxiety (across anxiety disorders) or measure a specific type of anxiety (e.g. panic disorder severity).

### Search strategy and selection criteria

**Fig. 1:**
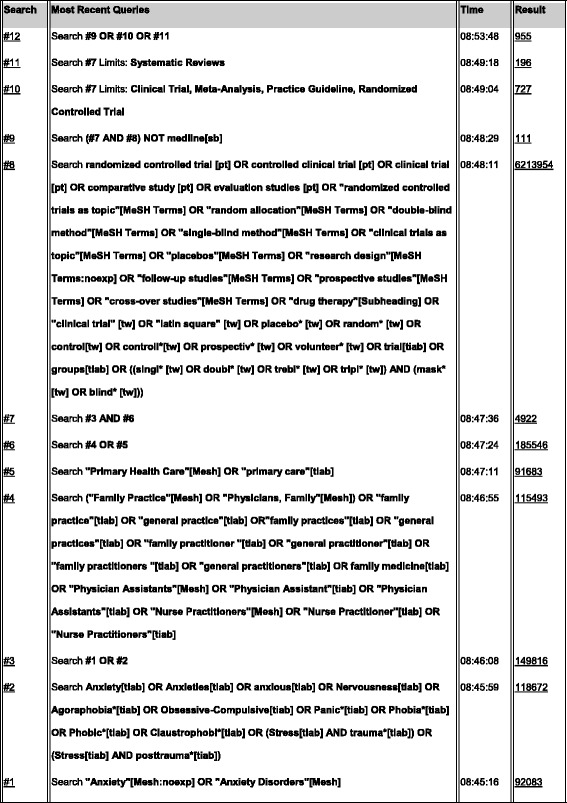
PubMed Search History for randomized controlled trials examining the effects of collaborative care for adult primary care patients with an anxiety disorder, compared to care as usual or another intervention

We searched PubMed (Medline), Psycinfo, Embase, The Cochrane Central Register of Controlled Trials, and Cinahl from inception to March 10^th^ 2014 without language restriction. The highly sensitive search was performed by an experienced librarian and author A.M., combining terms related to anxiety, primary care, and randomized controlled trials. For Medline, we used free text words and MeSH terms such as “Anxiety”, “Anxiety Disorders”, “Primary Health Care” and “Family Practice”. We combined these with a pre-tested search string for randomized controlled trials. See Fig. 1 for the full search strategy as performed in PubMed, which was adapted for use in the other databases. The reference lists of selected randomized controlled trials (RCTs) and reviews were checked for potentially relevant titles. The search was limited to published studies.

### Study selection

Titles and abstracts of retrieved studies were screened independently by two reviewers (AM/CFC) using a list of inclusion criteria. If a study appeared eligible (or if eligibility was doubtful), the full text of an article was retrieved. All full-text articles were assessed for eligibility by two independent reviewers (AM and AvB/HvM/CFC). Disagreement was resolved by consensus or a third reviewer. The outcome data were extracted by two reviewers independently (AM and CFC). Other relevant characteristics of studies were extracted by one author (AM) using a form based on Cochrane criteria [27] (see Table 1).

**Table 1:** Characteristics of randomized controlled trials comparing collaborative care for anxiety disorders with care as usual

Study No.	Authors/ year	De-sign	Recruit-ment	Diagn instr.	Int.	Setting	*N*(ITT)	Collaborative care intervention	Professionals involved	Comparison intervention	Outcomes FU	Outcome CC vs CAU	Outcome at 12 months [95 % CI]
1	Roy-Byrne et al. 2001 (Study 1)	RCT	Referral Screening (waiting room, PHQ-2 PD)	PD CIDI	CC vs CAU	3 primary care clinics (US)	CC: 57 CAU: 58	Medication management by psychiatrist	PCP, psychiatrist	CAU by PCP, i.e. pharmacotherapy or referral to mental health professional	ASI, PDSS 3,6,9,12 months	-Improved anxiety outcome at 3,6 and 12 months -Improved panic outcome at 6 months	-Anxiety/panic (ASI): T = 2.14, *p* = 0.035 ES 0.45 [0.03-0.87] -Panic (PDSS): statistics continuous outcome not reported
2	Roy-Byrne et al. 2005 (Study 2)	RCT	Referral Screening (waiting room, PHQ-2 PD)	PD CIDI	CC vs CAU	University affiliated primary care clinics (US)	CC: 119 CAU: 113	CBT and/or antidepressant medication	PCP, CM, psychiatrist	CAU by PCP, i.e. pharmacotherapy or referral to mental health professional	ASI 3,6,9,12 months	-Improved anxiety/panic outcome at all time points	- Anxiety/panic (ASI): Dif −6.64 [−10.73 to −2.48] *p* <0.001; ES 0.48 [0.18-0.78]
3	Rollman et al. 2005 (Study 3)	RCT	Screening (waiting room, PHQ)	PD/ GAD PRIME-MD	CC vs CAU	4 university affiliated primary care practices (US)	CC: 116 CC: 75	Guided selfhelp and/or antidepressant medication and/or referral to mental health specialist	PCP, CM, psychiatrist/ Psycho-therapist	CAU by PCP and patients received a diagnosis specific brochure	SIGH-A, PDSS 2,4,8,12 months	-Improved anxiety outcome at 12 months - Improved panic outcomes at 12 months -No sign. improvement in GAD outcomes	-Anxiety (SIGH-A): Dif −3.6 [ −6.4 to −0.8] *p* = 0.01; ES 0.43 [0.10-0.77] -Panic (PDSS): Diff −3.3 [−5.5 to −1.1] *p* = 0.004; ES 0.58 [0.19-0.97] -GAD (SIGH-A): Diff −1.1 [−5.0 to 2.7] *p* = 0.57; ES 0.13 [−0.32 to 0.58]
4	Konig et al. 2009 (Study 4)	Clus-ter RCT	Screening (PHQ)	PD/ GAD/ any AD PHQ	CC vs CAU	46 primary care practices (GER)	CC: 201 CAU: 188	Counselling (CBT) by the PCP	PCP, psychiatrist/ Psycho-therapist	CAU by PCP, including referral to mental health professional	BAI 6,9 months	- No difference in anxiety outcomes	-Anxiety (BAI): CC: M 18.18 SD 12.17 CAU: M 16.72 SD 10.34, *p* = 0.35; ES −0.13 [−0.36-0.10]
5	Roy-Byrne et al. 2010 (Study 5)	RCT	Referral	PD/ GAD/ SOP/ PTSD MINI	CC vs CAU	17 primary care clinics (US)	CC: 503 CAU: 501	CBT and/or antidepressant medication	PCP, CM, psychiatrist	CAU by PCP, i.e. medication, counseling or referral to mental health professional	BSI, PDSS, GADSS, SPIN, PCL 6,12,18 months	-Improved anxiety outcome at all time points -Improved panic outcome at 6 and 12 months -Improved GAD outcome at all time points -Improved SOP outcome at 6 and 12 months -No sign. improvement in PTSD outcomes	-Anxiety (BSI): Diff −2.63 [ −3.73 to −1.54] *p* <0.001; ES 0.33 [0.19-0.47] -Panic (PDSS): Diff −2.71 [ −4.29 to −1.14] *p* = .003; ES 0.48 [0.20-0.76] -GAD (GADSS): Diff −2.34 [−3.22 to −1.45] *p* <0.001; ES 0.49 [0.30 to 0.68] -SOP (SPIN): Diff −5.71 [−10.74 to −0.68] *p* = 0.08; ES 0.43 [0.05 to 0.81] - PTSD (PCL-C): Diff −7.7 [−17.55 to 2.15] *p* = 0.49; ES 0.45 [−0.12 to 1.02]
6	Oosterbaan et al. 2013 (Study 6)	Clus-ter RCT	Referral	PD/AGO/GAD/SOP/SP MINI	CC vs CAU	22 primary care practices (NL)	CC: 28 CAU: 27	Step 1) CBT based guided self-help with antidepressant medication for moderate disorder Step 2) CBT and medication in specialty care	PCP, CM, psychiatrist, CBT therapist	CAU by PCP, i.e. medication, counseling or referral to mental health professional	CGI-I, CGI-S, HRS-A 4,8,12 months	-Improved anxiety outcomes at 4 months	-Anxiety (HRS-A) CC: M 6.14 SD 5.26 CAU M: 8.11 SD 7.83 *p* = 0.02 ES 0.29 [−0.29 to 0.87]
7	Muntingh et al. 2014 (Study 7)	Clus-ter RCT	Referral Screening (PHQ)	PD/ GAD MINI	CC vs CAU	43 primary care practices (NL)	CC: 114 CAU: 66	Step 1) CBT based guided self-help Step 2) CBT Step 3) antidepressant medication	PCP, CM, psychiatrist, CBT therapist	CAU by PCP, i.e. medication, counseling or referral to mental health professional (including CM randomized to CAU)	BAI	-Improved anxiety outcome at all time points -Improved panic outcome at all time points -No sign. improvement in GAD outcomes	-Anxiety (BAI) Diff −6.84 [ −10.13 to −3.55] *p* <0.001; ES 0.73 [0.37-1.09] - PD (BAI): Diff −9.29 [−12.99 to −5.59] ES 1.03 [0.60 to 1.46] - GAD (BAI): Diff −1.13 [−7.33 to 5.08] *p* = 0.72 ES 0.13 [−0.56 – 0.81]

Abbreviations: *AD* anxiety disorder, *ASI* anxiety sensitivity index, *BAI* Beck Anxiety Inventory, *CAU* care as usual, *CBT* cognitive behavioral therapy, *CC* collaborative care, *CI* confidence interval, *CIDI* Composite International Diagnostic Interview, *CM* care manager, *ES* effect size, *GAD* generalized anxiety disorder, *GADSS* Generalized Anxiety Disorder Severity Scale, *GER* Germany, *HRS*-A Hamilton Rating Scale for Anxiety, *ITT* intention to treat, *MINI* Mini-International Neuropsychiatric Interview, *NL* Netherlands, *PD* Panic disorder, *PCL-C PTSD* Checklist–Civilian Version, *PCP* primary care physician, *PDSS* panic disorder severity scale, *PHQ* Patient Health Questionnaire, *PRIME-MD* Primary Care Evaluation of Mental Disorders, *PTSD* post traumatic stress disorder, *SIGH-A* Hamilton Anxiety Rating Scale, *SOP* social phobia, *SP* specific phobia, *SPIN* Social Phobia Inventory, *US* United States

### Data collection process

We extracted sample size, means, and standard deviations for scores on anxiety scales at baseline and follow-up for the intervention group and the control group. Outcomes for anxiety disorders in general as well as outcomes for specific anxiety disorders were extracted. For studies using more than one validated anxiety scale as an outcome measure, we chose the reported primary outcome measure. If the mean and standard deviation were not reported, we searched for other data necessary to calculate an effect size, such as a difference score with a standard deviation or confidence limits, and *p*-value [27]. Furthermore, data relevant for the interpretation of the findings such as the setting, diagnoses, the interventions used and the professionals involved were collected (see Table 1). Where published protocols of the studies included were available, they were used to supplement data about intervention details.

### Risk of bias in individual studies

The risk of bias of each included study was assessed using a standard form based on Cochrane criteria [28] by two reviewers (AM and AvB/HvM) independently. The form systematically enquired about possible sources of bias in randomized controlled trials, such as the adequacy of the randomization procedure, allocation concealment, handling of missing data and selective reporting. Disagreement between reviewers about assessment ratings were resolved by consensus or a third reviewer (CFC).

### Synthesis of results

We statistically summarized the effectiveness of collaborative care interventions versus the comparison interventions using meta-analysis. The analyses were conducted using the software package Comprehensive Meta Analysis version 2.0 [29]. We calculated a standardized mean difference (SMD) from reported differences in means on a continuous anxiety scale between interventions at 12 months follow-up. We summarized the SMDs using the random effects model [30]. To assess the heterogeneity among studies, we calculated the *I*^*2*^ statistic, which reflects the proportion of total variation across studies that is attributable to heterogeneity rather than chance. An *I*^*2*^ of 0 % means that there is no observed heterogeneity, while an *I*^*2*^ of 25, 50 and 75 % may be interpreted as low, medium, and high heterogeneity respectively [31].

### Risk of bias across studies

Funnel plots were created and Duval and Tweedie’s trim and fill method was used to examine the possibility of publication bias [32]. This method gives an estimate of the effect size after correcting for possible publication bias.

### Additional analyses

A predefined subgroup analysis was performed to assess the effectiveness of collaborative care for patients with a specific anxiety disorder. A disorder-specific outcome measure (if available) was used to calculate the effect size in a random effects meta-analysis. In an additional subgroup analysis, we examined several variables related to study procedures and intervention details based on the meta-regression of Coventry and colleagues [25]. We omitted variables described by Coventry and colleagues that were not present in any of the included studies and added the element of stepped care as this was a prominent aspect of studies 6 and 7 [21, 22]. For this subgroup analysis, we used a mixed-effects model which pools studies in a subgroup using a random effects model. In addition, we used a fixed-effects model to interpret variance between studies (Q and I^2^) and to test for significant differences between subgroups.

## Results

### Study selection

**Fig. 2:**
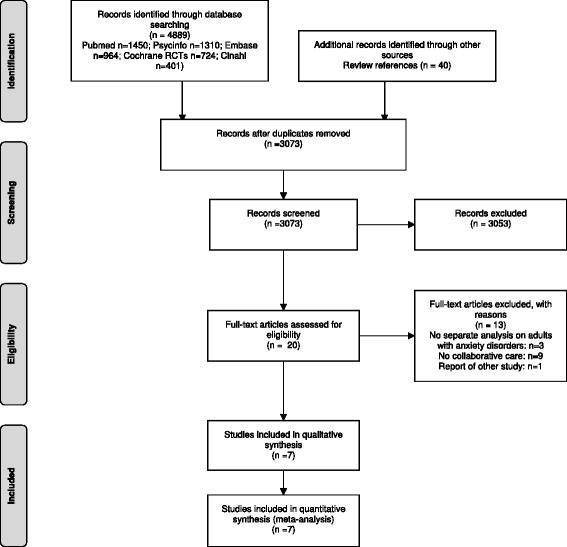
PRISMA flowchart [50]

The literature search resulted in a total of 4929 retrieved citations. After removal of duplicates, 3073 abstracts were available (see Fig. 2). Main reasons for exclusion of studies based on titles or abstracts were no RCT, no inclusion of anxiety disorders and no collaborative care. For 20 studies the full-text paper was retrieved and examined for inclusion. After the exclusion of 13 studies (nine no collaborative care; three no separate outcome reported for patients with anxiety disorders; one report of other study), there were seven studies that met all inclusion criteria.

### Study characteristics

Seven studies involving 2105 participants (1107 in the collaborative care condition, 998 in the control condition) were included: Roy-Byrne et al. 2001 [33] (study 1); Roy-Byrne et al. 2005 [34] (study 2); Rollman et al. 2005 [35] (study 3); König et al. 2009 [36] (study 4); Roy-Byrne et al. 2010 [37] (study 5); Oosterbaan et al. 2013 [21] (study 6); and Muntingh et al. 2014 [22] (study 7). Table 1 shows an overview of characteristics of the included studies.

#### Design and participants

The number of participants in each study ranged from 57 to 1004. Of the trials, four were individually randomized controlled trials (study 1–3, 5); three used cluster randomization on the level of primary care practices (study 4, 6, 7); four were conducted in the USA (studies 1–3,5), one in Germany (study 4) and two in the Netherlands (study 6 and 7). All studies reported that their study protocol was approved by the relevant medical ethical committee (Study 4, 6 and 7) or the institutional review board (study 1–3, and 5). Two studies exclusively included patients with panic disorder (studies 1 and 2), two studies (studies 3 and 7) included patients with panic disorder and/or generalized anxiety, and three studies (studies 4–6) included multiple anxiety disorders. Comorbid depression was allowed in all studies and was reported in five studies (Study 1–3, 5, 7), with prevalence rates ranging from 31 % (Study 7) to 64 % (Study 5). With one exception, studies used a structured interview to classify the anxiety disorder at baseline (CIDI, PRIME-MD). König and colleagues (study 4) determined the presence of an anxiety disorder by a cut-off score on the Patient Health Questionnaire. To recruit participants, two studies used screening only (studies 3 and 4), two studies (studies 5 and 6) used referral of primary care physicians, while three studies used both methods (studies 1,2,7).

#### Collaborative care and comparison interventions

The content of collaborative care interventions varied considerably between studies. In all studies, a primary care physician and a psychiatrist were involved, while in five studies a care manager was introduced as well (studies 2,3,5–7). One study used only medication management by a psychiatrist (study 1) and one study evaluated counselling with CBT elements by a trained primary care physician (study 4). The other studies used more comprehensive programs with a choice between, or combination of, antidepressant medication and CBT. Two studies (studies 6 and 7) used a stepped care program. Table 2 describes the characteristics of collaborative care in the included studies.

**Table 2:** Characteristics of collaborative care interventions for anxiety disorders

Study no.	Study (first author, year)	Professionals involved	Professional training	Interventions used	No. contacts with professionals	Collaboration between professionals	Monitoring	Follow-up / relapse prevention
1	Roy-Byrne et al. 2001	PCP Psychiatrist	PCP: 1-h didactic, medication algorithm	Medication management (paroxetine) and encouragement of adherence and exposure by psychiatrist Educational patient video	2 visits and 2 phone calls by psychiatrist	The PCP received a typed consultation note after each psychiatric visit.	No information provided	5 follow-up calls by psychiatrist
2	Roy-Byrne et al. 2005	PCP, Psychiatrist CM	PCP: 1-h didactic on, medication algorithm CM: treatment protocol, six videotapes, 1 day long workshop in care management and CBT	Face-to-face CBT Antidepressant medication according to algorithm by PCP Educational video and workbook	6 sessions by CM	Weekly caseload supervision of CM by psychiatrist 2-way communication of CM and PCP by telephone, fax, and e-mail. Recommendations as needed from a consulting psychiatrist to the PCP via the CM	No information provided	Intended were 6 telephone follow-up contacts during 10 months after the active treatment phase by CM
3	Rollman et al. 2005	PCP, Psychiatrist CM	PCP: 1-h conference and individual meeting of study investigators with PCPs, medication algorithm CM: study protocol and self-management workbooks, attending lectures at the University	CBT based guided self-help Antidepressant medication according to algorithm by PCP Referral to a community mental health specialist	7 telephone contacts by CM	Weekly caseload supervision of CM by psychiatrist Advice from the psychiatrist to the PCP and patient via CM Communication facilitated through an ambulatory EMR system	Monitoring by CM with PDSS / GADSS	Telephone contacts every 1–3 months after the acute phase to monitor symptoms
4	König et al.2009	PCP, Psychiatrist/ Clinical psychologist	PCP: 10 h training and two additional sessions on counseling skills and CBT	Counseling by PCP, including CBT techniques	No information provided	As needed consultation by psychiatrist/clinical psychologist at PCPs’ practices	No information provided	No information provided
5	Roy-Byrne et al. 2010	PCP, Psychiatrist CM	PCP: single-session training, medication algorithm CM: treatment protocol, 6 half days of didactics in care management and CBT	Face-to-face CBT by CM supported by computer program Antidepressant medication according to algorithm by PCP	6–8 sessions by CM	Weekly caseload supervision of CM by psychiatrist/psychologist “Regular” interaction between PCP and CM in person and by telephone As needed consultation of PCP by psychiatrist Communication facilitated through a webbased monitoring system	Monitoring with OASIS by CM	Monthly follow-up telephone calls by CM
6	Oosterbaan et al. 2013	PCP Psychiatrist/ CBT specialist CM	PCP: one educational session, medication algorithm CM: treatment protocol, 2-day training session in basic CBT strategies	Stepped care (according to severity): 1. CBT based guided self-help with support by CM (face-to-face) with antidepressant medication according to algorithm by PCP for patients with a moderate disorder 2. CBT and antidepressants in specialised mental health service	Step 1: 5 sessions by CM Step 2: No information provided	2-weekly supervision of CM by CBT specialist As needed consultation of PCP by psychiatrist	Monitoring with CGI by CM	No information provided
7	Muntingh et al. 2014	PCP Psychiatrist/ CBT specialist CM	PCP: 3 h workshop, medication algorithm CM: treatment protocol, 3-day workshop in care management and CBT strategies	Stepped care: 1. CBT based guided self-help with support by CM (face-to-face) 2. CBT by CM 3. Antidepressant medication according to algorithm by PCP	Step 1: 5 sessions by CM Step 2: 6 sessions by CM	Intended was 3-weekly supervision of CM by psychiatrist/ CBT specialist GP and CM were “instructed to frequently discuss treatment progress” As needed consultation of PCP by psychiatrist	Monitoring with BAI by CM	Monthly follow-up telephone calls by CM

Abbreviations: *CBT* cognitive behavioral therapy, *CM* care manager, *PCP* primary care phyisican

All studies compared the collaborative care intervention to usual care coordinated by the primary care physician. Table 3 gives an overview of the percentage of patients receiving pharmacotherapy, appropriate pharmacotherapy, counseling, CBT, and (specialized) mental healthcare as reported in the included studies. Pharmacotherapy was the most frequent treatment method reported in usual care.

**Table 3:** Care received in the collaborative care and care as usual conditions (*N* = 7)

Content of care*	Pharmaco-therapy (%)		Approriate pharmaco-therapy (%)		Counseling (%)		CBT (%)		Referral to mental health professional (%)	
Study	CC	CAU	CC	CAU	CC	CAU	CC	CAU	CC	CAU
Roy-Byrne et al. 2001*	77 %^a^	48 %^a^	47 %^b^	33 %^b^	NA	NA	NA	NA	NA	25 %
Roy-Byrne et al. 2005	54 %^c^	52 %^c^	41 %^b^	39 %^b^	70 %	34 %	63 %^d^	14 %^d^	NA	NA
Rollman et al. 2005	77 %^e^	66 %^e^	NA	NA	79 %^f^	NA	66 %^g^	NA	18 %	26 %
König et al. 2009	NA	NA	NA	NA	NA	NA	NA	NA	33 %	33 %
Roy-Byrne et al. 2010	70 %^h^	68 %^h^	46 %^i^	42 %^i^	88 %	51 %	82 %^j^	34%^j^	NA	NA
Oosterbaan et al. 2013*	45 %	33 %	NA	NA	NA	NA	75 %^g^	NA	NA	NA
Muntingh et al. 2014	21 %^e^	35 %^e^	NA	NA	92 %	12 %	78 %^g^	NA	11 %	21 %

*Highest % of patients that have received a form of care at any follow-up measurement

^a^Appropriate type of medication

^b^Adequate dose and duration of medication

^c^ Any antipanic pharmacotherapy

^d^3 or more sessions counseling plus at least 4 of 7 CBT techniques

^e^SSRI/SNRI pharmacotherapy

^f^3 or more telephone contacts with CM

^g^3 or more (telephone) contacts with CM about CBT workbook

^h^Any psychotropic medication

^I^Appropriate type, dose and duration

^j^Counseling with at least 3 CBT elements

*NA* = Not Available

#### Outcome measures

All studies included a continuous outcome scale to measure anxiety. Study 2 used a dichotomous outcome (remission and response) as primary outcome measure. Five studies reported separately about panic disorder outcomes (studies 1–3,5,7). The length of follow-up varied from 9 months (study 4) to 18 months (study 5). Two studies (studies 4 and 7) used patient self-report to assess the outcome, while in the other five studies a (blinded) research assistant administered the outcome measures by telephone.

### Risk of bias within studies

The overall quality of included studies was moderate to high (see Table 4). For study 1, six out of eight criteria were rated as unclear; in the other studies four to six criteria were rated as low risk of bias. The most prevalent potential source of bias was the inability to blind patients and professionals for treatment allocation, as is common in psychotherapy research [38]. Studies 6 and 7 used cluster randomization with subsequent referral by the primary care physician, which may have induced selection bias. Study 1 did not provide the statistics of an insignificant result on the Panic Disorder Severity Scale (PDSS). Of four studies (studies 2,3,5, 7), a published study protocol was retrieved [39–42].

**Table 4:** Risk of bias in 7 randomized controlled trials comparing collaborative care for adult patients with anxiety disorders to usual primary care

	Adequate Sequence Generation?	Allocation concealed?	Patients blinded?	Professionals blinded?	Outcome assessors blinded?	Incomplete outcome data addressed?	Free of selective reporting?	Free of other bias
Roy-Byrne et al. 2001	+	?	?	?	+	?	?	?
Roy-Byrne et al. 2005	?	+	–	–	+	+	?	+
Rollman et al. 2005	+	+	–	–	+	+	?	+
König et al. 2009	+	+	–	–	?	+	?	+
Roy–Byrne et al. 2010	+	+	?	–	+	+	+	+
Oosterbaan et al. 2013	+	+	–	–	+	+	+	+
Muntingh et al. 2014	+	+	–	–	+	+	+	+

### Results of individual studies

**Fig. 3:**
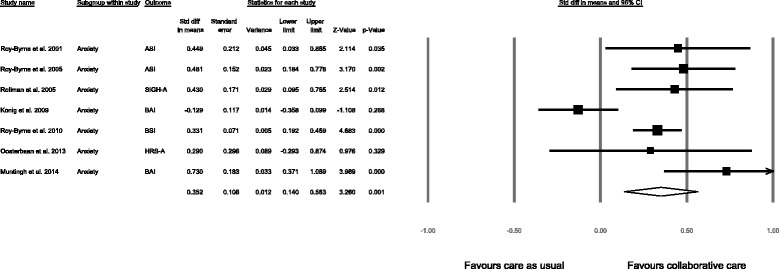
Meta-analysis for the effect of collaborative care vs. care as usual on continuous anxiety scales at 12 months follow-up

The results of the individual studies at follow-up closest to 12 months are reported in Table 1. All studies except study 4 reported a significantly greater effect of the collaborative care intervention compared to care as usual. The meta-analysis (Fig. 3), pooling the data of all seven studies, yielded an SMD of 0.35 (95 % CI 0.14 to 0.56, *p* = 0.001), indicating that collaborative care leads to a significantly greater reduction in anxiety symptoms, with a small effect size after 12 months. The *Q*-value was 21.73 (df = 6, *p* = 0.001), indicating significant dispersion across studies. The *I*^2^ was 72 %, which indicates that a high proportion of the total variation may be attributed to true heterogeneity between studies. A sensitivity analysis excluding Study 4 led to a considerable decrease in heterogeneity (*Q* = 4.72, df = 5, *p* = 0.34; *I*^*2*^ = 0 %) and a higher SMD (SMD = 0.40, 95 % CI 0.30 to 0.51, *p* < 0.001).

### Risk of bias across studies

The funnel plot showed an indication (not significant) for publication bias for the analysis on all anxiety disorders (Egger’s test, two-tailed *p* = 0.57). According to the trim and fill method [32], the SMD should be adjusted from 0.35 to 0.25 (95 % CI 0.16 to 0.34). This asymmetry may indicate that the effect of collaborative care for anxiety disorders is slightly overestimated due to publication bias, although asymmetry may also be attributed to true heterogeneity between studies [43]. Indeed, heterogeneity was high (*I*^2^ = 72 %), which may be attributed to differences between the three European studies (*I*^2^ = 78 % in European studies vs. *I*^2 =^ 0 % in US studies). For the subgroup analysis on panic disorder, no indication was found for a publication bias (Egger’s test, two-tailed *p* = 0.29). Furthermore, we ran a sensitivity analysis excluding the lowest quality study (Study 1). This had no significant effect on the analysis on anxiety disorders (SMD_adjusted_ 0.34, 95 % CI 0.10 – 0.58) or panic disorder (SMD_adjusted_ 0.60, 95 % CI 0.38 to 0.83).

### Additional analysis: disorder-specific impact of collaborative care

**Fig. 4:**
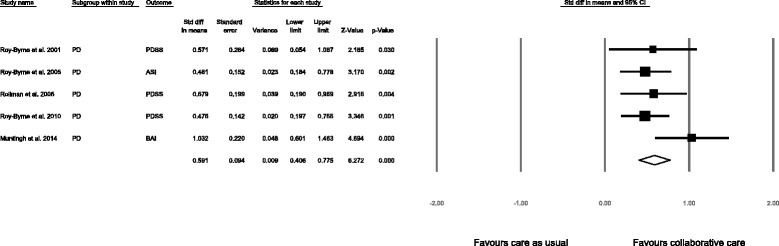
Subgroup-analysis for the effect of collaborative care vs. care as usual at 12 months follow-up for patients with a panic disorder

A predefined subgroup analysis was performed for patients with panic disorder. Outcomes on the Panic Disorder Severity Scale (PDSS) were used when reported. For the two studies (2,7) that did not report the PDSS, the primary outcome measure was used in the meta-analysis. Because Roy-Byrne and colleagues [33] reported an insignificant result on the PDSS, but were not able to provide the statistics necessary for calculating the effect size, we used the ASI score. The combined effect size of the four studies comparing collaborative care to care as usual in patients with panic disorder was 0.59 (95 % CI 0.41 to 0.78, *p* < 0.001), which may be interpreted as a moderate effect size (Fig. 4).

Concerning anxiety disorders other than panic disorder, studies 3 and 7 reported no significant effect for patients with generalized anxiety disorder only (Table 1). Study 5 reported a significant difference between collaborative care and care as usual on the Brief Symptom Inventory (BSI) for panic disorder, generalized anxiety disorder, and social phobia, but not for posttraumatic stress disorder (PTSD). In a subsequent report on this study [44], disorder-specific data on specific scales were reported, with similar results (Table 1).

### Additional analysis: variables related to outcome

Additional analyses were performed to assess the impact on outcome of variables related to study procedure and content of the intervention (see [25]; Table 5). The analysis revealed that studies performed in the United States were more homogeneous (*I*^*2*^ = 0 % vs. 87 %) and on average yielded a significantly greater effect size than studies performed in European countries (SMD 0.37 vs. 0.29, *p* = 0.03). Furthermore, the five studies that included a care manager had a significantly higher effect size (SMD 0.42 vs. 0.13, *p* = 0.001) than those without. Lastly, the two studies using stepped collaborative care (both from the Netherlands) yielded a greater effect size than studies that did not use stepped care (SMD 0.57 vs. 0.29, *p* = 0.04). However, these analyses need to be interpreted with caution due to the small number of studies and the considerable heterogeneity between European studies.

**Table 5:** Meta-analysis with between-study subgroup analyses of variables related to study procedures and content of collaborative care

Covariate	Number	SDM	95% CI	Q	I^2^	*P*
All studies	7	0.35	0.14 – 0.56	21.73	72.39	
Country						0.031*
US	4	0.37	0.26 – 0.49	1.10	0.00	
European	3	0.29	−0.31 – 0.89	15.98	87.50	
Randomization procedure						0.031*
Patient randomization	4	0.37	0.26 – 0.49	1.10	0.00	
Cluster randomization	3	0.29	−0.31 – 0.89	15.98	87.50	
Recruitment method						0.65
Referral by professional	2	0.33	0.19 – 0.47	0.02	0.00	
Systematic identification (or both)	5	0.38	0.05 – 0.71	21.51	81.41	
Care manager						0.001*
Care manager	5	0.42	0.29 – 0.55	4.66	14.24	
No care manager	2	0.13	−0.43 – 0.70	5.69	82.43	
Intervention content						0.82
Psychological intervention (CBT) with/without medication management	5	0.42	0.29 – 0.55	4.66	14.24	
Medication management alone	1	0.45	0.03 – 0.87	–	–	
Not applicable	1	–	–	–	–	
Stepped care						0.041*
Stepped care	2	0.57	0.06 – 0.53	1.58	36.85	
No stepped care	5	0.29	0.16 – 0.99	15.97	74.95	
Supervision frequency specialist-care manager						0.056
Ad hoc	1	0.73	0.37 – 1.09	–	–	
Scheduled (i.e. at least 2-weekly)	4	0.36	0.25 – 0.48	1.03	0.00	
Not applicable	2	–	–	–	–	

*p<0.05

## Discussion

### Summary of evidence

The results of this meta-analysis indicate that collaborative care is more effective than usual primary care at twelve months follow-up, with a small effect size for all anxiety disorders combined (SDM 0.35) and a moderate effect size for patients with panic disorder (SMD 0.59). The quality of studies was moderate to high. There were indications for considerable heterogeneity between European studies in contrast to studies performed in the United States.

### Strengths and limitations

A strength of this study is that we conducted this review using PRISMA criteria [26]. Furthermore, we were able to examine the long-term effects of collaborative care, which is often problematic in reviewing effectiveness studies on anxiety disorders in primary care [45]. However, there are several limitations in this systematic review and meta-analysis that need consideration.

We identified only seven studies that met our inclusion criteria. As our search was limited to published articles, we probably have missed (unpublished) RCTs, which generally report lower effect sizes [46]. For example, we omitted a study by Rollman and colleagues [47], including 250 patients with generalized anxiety disorder or panic disorder, because their results remain unpublished. They reported an effect size of 0.30 in a conference abstract, which is comparable to the overall effect size found in our meta-analysis.

Furthermore, some methodological issues should be mentioned. One study [33] did not report the statistics for the insignificant effect on the disorder-specific measure. However, a sensitivity analysis excluding this study did not alter the results. Also, two studies [21, 22] used referral as a recruitment method after cluster randomization, which may cause selection bias and inflated effect sizes [48]. Yet Muntingh and colleagues, who used both referral and screening to recruit patients, reported nearly equal effect sizes in a subgroup analysis on patients selected by screening [22]. Lastly, one may debate on the broad concept of collaborative care. The seven studies that fulfilled our inclusion criteria varied on several characteristics, such as the inclusion of a care manager, care manager supervision, provision of CBT, and following a stepped care protocol. Common elements in all seven studies were the input of specialist mental health in primary care, collaboration between professionals and the provision of evidence based interventions. As we are coming closer to defining the most effective elements of collaborative care (such as the provision of psychological treatment by a care manager [25]), the concept of collaborative care can be refined in the future, leading to more homogeneous results.

### Comparison with the literature

The results from our meta-analysis compare to those reported in previous meta-analyses on collaborative care for anxiety disorders [19, 20] and extend their results with the inclusion of recent European and collaborative stepped care studies. The SMD of 0.35 found in our meta-analysis is also similar to the SMD of 0.28 which was reported in two large meta-analyses about collaborative care for depressive disorders [19, 25].

Comorbid depression was prevalent in the included studies (31–64 %). Unfortunately, none of the studies reported on the effects of comorbid depression on outcome, while depression has been related to a poor outcome of treatment for anxiety disorders [49].

We found that studies performed in the US had a greater effect size compared to European studies, which is in contrast with previous findings [25]. The US studies were also considerably more homogeneous than the European studies, which may be related to similarities and differences in healthcare systems between the US and Europe [16]. The studies in the US were mainly conducted in large clinics (often university-affiliated) using specifically trained and employed care managers, which may have facilitated effective implementation of the intervention, while the European studies were performed in more diverse settings in rather small primary care practices with collaborating professionals who were only involved in collaborative care on a part-time basis [21]. However, the heterogeneity between the European studies may also be related by other study characteristics such as design and intervention details. In fact, the study of König and colleagues [36] was the only study to report a non-significant effect of the intervention, and excluding this study from the analysis led to a considerable decrease in heterogeneity. The absence of a significant effect of this study could be related to a) suboptimal implementation and b) the fact that they employed the most ‘basic’ package of collaborative care. Both careful implementation and the application of a comprehensive collaborative care program may thus be crucial to the effectiveness of collaborative care. Including the study of König and colleagues probably provides a conservative estimate of the effects of collaborative care for anxiety disorders. In any case, compared to psychological treatment alone for anxiety disorders in primary care [45], the results of collaborative care are promising.

### Implications for research

Most importantly, more randomized controlled trials and, subsequently, individual patient data meta-analyses are warranted to better identify who can benefit from what aspect of the intervention. We found a somewhat greater effect size for patients with panic disorder than for all anxiety disorders combined. In fact, evidence for the effectiveness of collaborative care for generalized anxiety disorders based on three included studies was inconclusive [21, 35, 37], so future research should address the effectiveness of collaborative care for anxiety disorders other than panic disorder. Furthermore, as described above, successful implementation of the intervention seems essential for the effectiveness of collaborative care. Hence, implementation should also be a focus of research on collaborative care. Lastly, cost-effectiveness analyses are needed to increase our knowledge on the benefits of collaborative care.

## Conclusions

This meta-analysis indicates that collaborative care is more effective than usual primary care for adult patients with common anxiety disorders (small effect size), and panic disorder in particular (moderate effect size). Since it is difficult to improve primary care for anxiety disorders, this is a promising result. More research is needed to increase diagnostic precision, disentangle elements that make collaborative care most effective, and to evaluate the effectiveness of collaborative care in different anxiety disorders.

## Abbreviations

AD; anxiety disorder, ASI; Anxiety Sensitivity Index, BAI; beck anxiety inventory, BSI; Brief Symptom Inventory, CAU; care as usual, CBT; cognitive behavioral therapy, CC; collaborative care, CI; confidence interval, CIDI; Composite international diagnostic interview, CM; care manager, ES; effect size, GAD; generalized anxiety disorder, GADSS; generalized anxiety disorder severity scale, GER; Germany, HRS-A; Hamilton rating scale for anxiety, ITT; intention to treat, MINI; mini-international neuropsychiatric interview, NL; Netherlands, PCL-C; PTSD checklist–civilian version, PCP; primary care physician, PD; Panic disorder, PDSS; panic disorder severity scale, PHQ; patient health questionnaire, PRIME-MD; primary care evaluation of mental disorders, PTSD; posttraumatic stress disorder, RCT; randomized controlled trial, SIGH-A; Hamilton anxiety rating scale, SMD; standardized mean difference, SOP; social phobia, SP; specific phobia, SPIN; social phobia inventory, US; United States.
